# Adaptation of the group A *Streptococcus* adhesin Scl1 to bind fibronectin type III repeats within wound‐associated extracellular matrix: implications for cancer therapy

**DOI:** 10.1111/mmi.14317

**Published:** 2019-06-12

**Authors:** Dudley H. McNitt, Soo Jeon Choi, Jessica L. Allen, River A. Hames, Scott A. Weed, Livingston Van De Water, Rita Berisio, Slawomir Lukomski

**Affiliations:** ^1^ Department of Microbiology, Immunology, and Cell Biology West Virginia University School of Medicine Morgantown WV USA; ^2^ Department of Biochemistry, Program in Cancer Cell Biology West Virginia University School of Medicine Morgantown WV USA; ^3^ Departments of Surgery and Regenerative and Cancer Cell Biology Albany Medical College Albany NY USA; ^4^ Institute of Biostructures and Bioimaging National Research Council Naples Italy

## Abstract

The human‐adapted pathogen group A *Streptococcus* (GAS) utilizes wounds as portals of entry into host tissue, wherein surface adhesins interact with the extracellular matrix, enabling bacterial colonization. The streptococcal collagen‐like protein 1 (Scl1) is a major adhesin of GAS that selectively binds to two fibronectin type III (FnIII) repeats within cellular fibronectin, specifically the alternatively spliced extra domains A and B, and the FnIII repeats within tenascin‐C. Binding to FnIII repeats was mediated through conserved structural determinants present within the Scl1 globular domain and facilitated GAS adherence and biofilm formation. Isoforms of cellular fibronectin that contain extra domains A and B, as well as tenascin‐C, are present for several days in the wound extracellular matrix. Scl1‐FnIII binding is therefore an example of GAS adaptation to the host's wound environment. Similarly, cellular fibronectin isoforms and tenascin‐C are present in the tumor microenvironment. Consistent with this, FnIII repeats mediate GAS attachment to and enhancement of biofilm formation on matrices deposited by cancer‐associated fibroblasts and osteosarcoma cells. These data collectively support the premise for utilization of the Scl1‐FnIII interaction as a novel method of anti‐neoplastic targeting in the tumor microenvironment.

## Introduction

Group A *Streptococcus* (GAS or *Streptococcus pyogenes*) is an obligate human pathogen that is responsible for over 700 million infections worldwide each year (Carapetis *et al.*, 2005). Infection by GAS can result in diseases that range in severity, from highly prevalent superficial infections to fatal conditions (Carapetis *et al.*, 2005). GAS infection can also lead to the development of post‐infectious autoimmune sequelae (Bisno *et al.*, 1970; Swedo *et al.*, 1997; Cunningham, 2000). Invasive infections and autoimmune sequelae result in over 500,000 deaths globally each year (Carapetis *et al.*, 2005; Ralph and Carapetis, 2013), placing GAS among the top 10 most lethal bacteria (Ralph and Carapetis, 2013). Superficial GAS infections account for the majority (~95.5%) of infections, mostly affecting children; GAS also asymptomatically colonizes the throat and skin of 5–25% of children of the general population (Efstratiou and Lamagni, 2016). GAS isolates are subtyped based on sequence polymorphisms within the 5′‐hypervariable end of the *emm* gene, encoding the N‐terminus of the M‐protein, a major surface adhesin and virulence factor of GAS (Fischetti, 2016); there have been over 220 identified *emm* types of GAS (Sanderson‐Smith *et al.*, 2014).

GAS strains express numerous adhesins that contribute to host colonization (Walker *et al.*, 2014). Two ubiquitous surface‐associated proteins are the streptococcal collagen‐like protein 1 (Scl1/SclA) and 2 (Scl2/SclB) (Lukomski *et al.*, 2000; 2001; Rasmussen *et al.*, 2000; Rasmussen and Björck, 2001; Whatmore, 2001). Both Scl1 and Scl2 are homotrimeric and share a distinct ‘lollipop‐like’ structural organization (Xu *et al.*, 2002; Han *et al.*, 2006b). The N‐terminal sequence‐variable (V) globular domain is followed by the collagen‐like (CL) domain, followed by a cell‐associated domain containing a cell wall anchoring LPATG motif at the C‐terminus (Lukomski *et al.*, 2000; 2001). The amino acid sequences of the V‐domains diverge between Scl1 and Scl2 proteins, as well as between Scl1 and Scl2 variants from strains of different M types (Lukomski *et al.*, 2017). The V‐domain of the Scl2 protein from M3‐type GAS has been crystalized and displays a six‐helix bundle fold, with two antiparallel α‐helices in each monomer, joined by surface‐exposed loops (Squeglia *et al.*, 2013; 2014; McNitt *et al.*, 2018). The surface‐exposed loops of the Scl1 V‐domain bind selected isoforms of cellular fibronectin (cFn) that are expressed within wounded tissue (Caswell *et al.*, 2010; Oliver‐Kozup *et al.*, 2013; McNitt *et al.*, 2018).

cFn is a high molecular weight glycoprotein encoded by the *FN1* gene that contains three different types of repeats (I, II and III) (Hynes, 1990; Ffrench‐Constant, 1995). There are over 20 different isoforms of cFn in humans due to alternative splicing of *FN1*‐mRNA, which can lead to the inclusion of fibronectin type III (FnIII) repeats, known as extra domain A (EDA/EIIIA) and extra domain B (EDB/EIIIB) and portions of the non‐homologous V or CSIII region (To and Midwood, 2011). The EDA and EDB repeats vary in sequence but retain the conserved prototypical FnIII‐repeat β‐sandwich structure, comprised of 7 β‐strands (A, B, C, C′, E, F and G) connected by flexible loops (Leahy *et al.*, 1996; Niimi *et al.*, 2001). Spatial and temporal inclusion of EDA and EDB domains in cFn is tightly regulated; both are expressed during embryogenesis but are not readily detectable in healthy adult tissue (Ffrench‐Constant and Hynes, 1989; Oyama *et al.*, 1989). Conversely, isoforms of cFn that contain EDA and/or EDB are expressed in pathological adult tissues, including wound beds and solid tumors (Zardi *et al.*, 1987; Ffrench‐Constant *et al.*, 1989; Singh *et al.*, 2004). EDA has been shown to play important roles in wound healing (Muro *et al.*, 2003; Longmate *et al.*, 2017). The role of EDB/cFn in the wound microenvironment is not well understood but is known to be expressed during healing‐ and tumor based angiogenesis (Castellani *et al.*, 1994; Birchler *et al.*, 2003; Gopal *et al.*, 2017). Tenascin‐C (TnC) is another ECM protein that contains canonical FnIII repeats (Midwood and Orend, 2009), and modulates cellular adhesin to other ECM proteins, and is bound directly by several cellular integrin receptors (Gulcher *et al.*, 1989; Tucker and Chiquet‐Ehrismann, 2015; Giblin and Midwood, 2015). TnC is predominately expressed during fetal development, with negligible levels found within normal adult soft tissue (Sahlberg *et al.*, 2001; Karus *et al.*, 2011; Chiquet‐Ehrismann *et al.*, 2014). TnC deposition is dramatically increased in adult wounds and is distributed within the stroma of solid tumors (Chiquet‐Ehrismann *et al.*, 1986; Midwood and Orend, 2009).

We previously reported that Scl1 binds to the wound‐associated fibronectin type III repeat, EDA, facilitating GAS colonization and biofilm formation on EDA/cFn coating and on matrices deposited by normal human dermal fibroblasts (Oliver‐Kozup *et al.*, 2013). In this report, we show that Scl1 binds to recombinant EDB and facilitates GAS biofilm formation on rEDB coatings. Similarly, Scl1 binds TnC and to the FnIII repeats of TnC. The Scl1‐V‐domain loop‐region was shown to be important in the recognition of both rEDB and recombinant FnIII repeats of TnC (rTnFnIII), and was conserved across phylogenetically distant Scl1 variants, originating from strains of epidemiologically relevant M types. In addition, Scl1 mediates GAS attachment and biofilm formation on ECM deposited by cancer‐associated fibroblasts and osteosarcoma cells that contain cFn isoforms with EDA and/or EDB, and TnC. This work identifies novel wound‐associated ECM targets for Scl1 that are expressed at the portal of entry for GAS and facilitate host colonization at wound sites, and implies the utilization of rScl1 in cancer therapy.

## Results

### Scl1 binds to EDB of cFn via surface exposed loops

We have shown that Scl1 binds to cFn, but not plasma fibronectin (pFn) (Caswell *et al.*, 2010), through direct binding to EDA (Oliver‐Kozup *et al.*, 2013). Interestingly, we determined that the commercial cFn preparations we used in binding assays contained cFn that included the EDB segment (data not shown). Therefore, we hypothesized that Scl1 binds to EDB, and that binding occurs by a similar mechanism as to EDA involving the surface‐exposed loops of the Scl1‐V domain (McNitt *et al.*, 2018).

**Figure 1:**
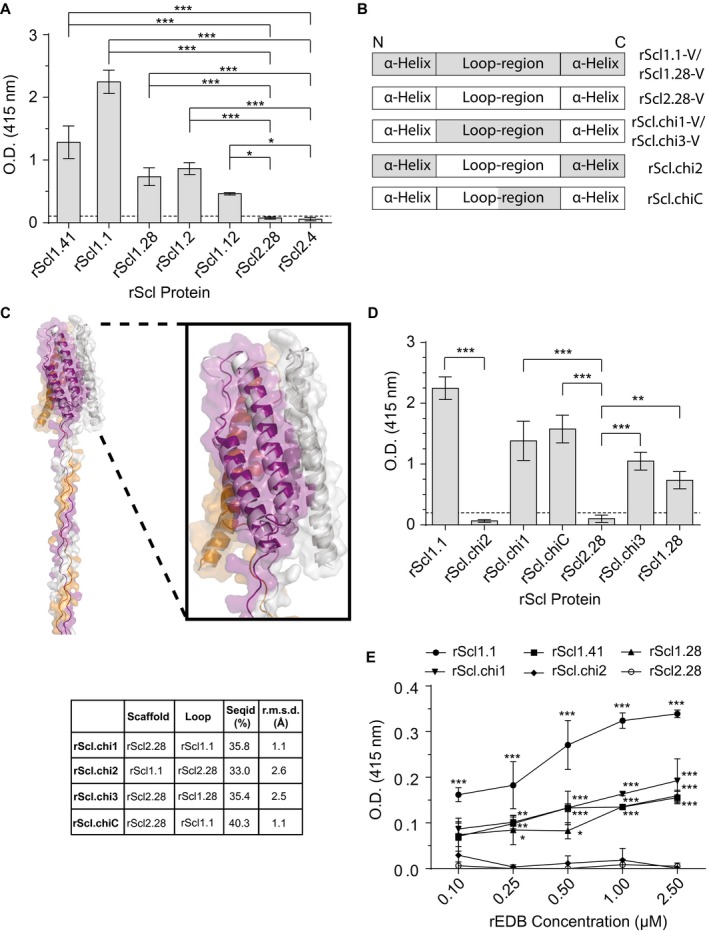
Scl1‐V domain binds fibronectin type III repeat, extra domain B (EDB), via surface‐exposed loops. Recombinant extra domain B (rEDB) was tested for binding to recombinant streptococcal collagen‐like proteins (rScl). A. rEDB binding to Scl1‐ and Scl2‐derived rScl constructs. rScl proteins were immobilized onto *Strep*‐Tactin‐coated microplate wells and incubated with rEDB. Primary anti‐His‐tag mAb and HRP‐conjugated secondary Ab were used for ligand detection by ELISA. Graph bars indicate the mean OD_415nm_ normalized against BSA controls. Statistical analysis was calculated using a one‐way ANOVA, from three independent experiments, each performed in triplicate wells (*N* = 3 ± SD); *P ≤ 0.05, ***P ≤ 0.001. Statistical significance evaluates the differences in rEDB binding by rScl1 proteins, as compared to ECM‐binding negative rScl2.28 and rScl2.4 control proteins. Dashed line indicates threshold OD_415nm_ +2SD values recorded for binding‐negative rScl2.28 control protein. B. Schematic representation of the variable (V) domains in recombinant Scl constructs used. Homotrimeric rScl1.1‐ and rScl1.28‐V domains (gray box), and rScl2.28‐V domain (white box) each consists of three conserved pairs of anti‐parallel α‐helices, with interconnecting loops (McNitt et al., 2018). Chimeric proteins were generated by replacing either the entire (rScl.chi1‐3) or partial (rScl.chiC) loop sequences between different constructs. C. I‐TASSER modeling of chimeric rScl proteins. *Left*, far‐out view of a representative I‐TASSER model of Scl.chi1, including the V domain and the first 16 triplets of the CL domain. *Right insert*, close‐up view of the Scl.chi1‐V domain. The three monomers are colored purple, orange and gray in both models. In close‐up view, white depicts Scl2.28, the loop‐host Scl protein of Scl.chi1. *Bottom*, I‐TASSER model sequence identities and root mean square deviations from Scl2.3, room mean square deviations performed using DALI server. D. rEDB binding to chimeric rScl constructs. ELISA was performed as described in panel A. Statistical analysis was calculated using a one‐way ANOVA, from three independent experiments, each performed in triplicate wells (*N* = 3 ± SD); **P ≤ 0.01, ***P ≤ 0.001. Statistical significance evaluates the differences in rEDB binding between chimeric proteins and their respective loop‐hosts: rScl.chi1, rScl.chiC and rScl.chi3 compared to binding‐negative control protein rScl2.28, and rScl.chi2 to binding‐positive control protein rScl1.1. Dashed line indicates threshold OD_415nm_ +2SD values recorded for binding‐negative rScl2.28 control protein. E. Concentration‐dependent binding of rEDB to rScl proteins. rScl proteins, immobilized onto *Strep*‐Tactin coated microplate wells, were incubated with increasing concentrations of rEDB (0.1–2.5 µM) and detected by ELISA, as described above. Statistical analysis was calculated using a two‐way ANOVA, from three independent experiments, each performed in triplicate wells (*N* = 3 ± SD); *P ≤ 0.05, **P ≤ 0.01, ***P ≤ 0.001. Statistical significance evaluates the differences in rEDB binding by rScl1 proteins, rScl.chi1 and rScl.chi2, as compared with ECM‐binding negative rScl2.28 control protein. [Colour figure can be viewed at wileyonlinelibrary.com]

We first investigated rEDB binding to an extended panel of rScl1 and rScl2 constructs, derived from diverse Scl1 and Scl2 variants that are found in strains M1, M2, M4, M12, M28 and M41, previously screened for binding to cFn (Caswell *et al.*, 2010), and rEDA (Oliver‐Kozup *et al.*, 2013). All cFn and rEDA‐binding rScl1 proteins demonstrated significant binding to rEDB, whereas control cFn‐ and rEDA‐binding‐negative rScl2 proteins also do not bind rEDB (Fig. 1A).

To determine the region of the Scl1‐V domain responsible for rEDB binding, we employed a panel of chimeric rScl constructs (Fig. 1B), previously shown to facilitate EDA recognition (McNitt *et al.*, 2018). Replacement of the 22‐amino‐acid loop sequence of the EDB‐binding‐negative rScl2.28‐V domain with the corresponding EDB‐binding‐positive rScl1.1 sequence (Scl1 of M1 strain), resulted in the rScl.chimera1 (rScl.chi1). Conversely, replacement of loop sequence in the rScl1.1‐V domain with the loop from rScl2.28‐V domain resulted in rScl.chi2. Two additional chimeric mutants were developed: (i) a partial loop substitution of the C‐terminal 11 amino acids of the loop from Scl1.1 that replaced the analogous loop sequence of rScl2.28, termed rScl.chiC, (ii) replacement of the entire 22‐amino‐acid loop sequence of rScl2.28 with the loop sequence from a phylogenetically distant EDB‐binding‐positive Scl1 variant, rScl1.28, termed rScl.chi3. We performed I‐TASSER (Iterative Threading Assembly Refinement) analysis to predict the impact of each loop substitution on the structures of rScl proteins. The conformation of all Scl‐chimeras differ only in the inserted loops (Table S1), whereas the overall fold of the molecules remains conserved, as shown in a representative I‐TASSER model of rScl.chi1 (Fig. 1C). Superposition of each chimera on the crystal structure of Scl2.3 produced overall root mean square values, computed on Cα atoms, in the range from 1.0 to 2.6 Å (Fig. 1C, *table*), ensuring that the replacement of each loop in the Scl‐V domain did not impact the overall structures of rScl proteins.

Chimeric proteins rScl.chi1‐3 and rScl.chiC were tested for rEDB‐binding by ELISA to evaluate the gain or loss of binding function, as a result of loop replacements (Fig. 1D). There was a significant gain of rEDB‐binding function, when the entire 22‐aa or the C‐terminal 11‐aa‐loop sequences from the rEDB‐binding positive rScl1.1 construct were transplanted into the rEDB‐binding negative background (rScl2.28), as detected for rScl.chi1 and rScl.chiC chimeric proteins. These results were further supported by a significant loss of rEDB‐binding by rScl1.1, observed for the rScl.chi2 construct harboring a reverse loop substitution from the rEDB‐binding negative rScl2.28 (Fig. 1D). A gain of rEDB‐binding function, similar to rScl.chi1, was detected for rScl.chi3 construct following the transfer of the V‐domain loop sequence from the rScl1.28 variant into rEDB‐binding‐negative background. Therefore, the Scl1‐V domain loop‐region is responsible for the recognition of rEDB, similar to what was reported for rEDA (McNitt *et al.*, 2018). The partial restoration of binding in both rScl.chi1 and rScl.chiC compared to Scl1.1 may be due to differences in the accessibility of the loop‐region for binding when it is within the Scl1‐V domain versus the Scl2‐V domain. A similar rationale may also explain the differences in binding between Scl.chi3 and Scl1.28.

Incubation of immobilized rScl1 proteins (rScl1.1, rScl1.28 and rScl1.41) and rScl.chi1 with increasing concentrations (0.1–2.5 µM) of rEDB demonstrates concentration‐dependent binding (Fig. 1E), approaching detection plateaus around 1–2.5 µM of rEDB. Conversely, rScl2.28 and rScl.chi2 did not demonstrate any increase in rEDB detection within the same concentration range, providing further validation that these two rScl proteins do not bind rEDB. These data reveal that the Scl1 adhesin binds to both the EDA and EDB segments that are found within cFn, expressed in the wounded portal of entry and signify the importance of the Scl1‐V loop in EDB/EDA recognition and binding.

### Scl1 mediates GAS‐EDB binding and promotes biofilm formation

We next determined if GAS‐expressed Scl1 mediates rEDB binding. We assessed GAS‐rEDB binding using M1 and M41 type GAS. M1 is a global pandemic M type, isolated in both non‐invasive and invasive infections (Nasser *et al.*, 2014), whereas M41 strains have historically been associated with impetigo (Top *et al.*, 1967; Wannamaker, 1970). M1 and M41 WT strains form biofilms *in vitro* (Oliver‐Kozup *et al.*, 2011; 2013) and Scl1 in each of these M types contributes to GAS infection *in vivo* (Lukomski *et al.*, 2000; Dohrmann *et al.*, 2014; Bachert *et al.*, 2016).

**Figure 2:**
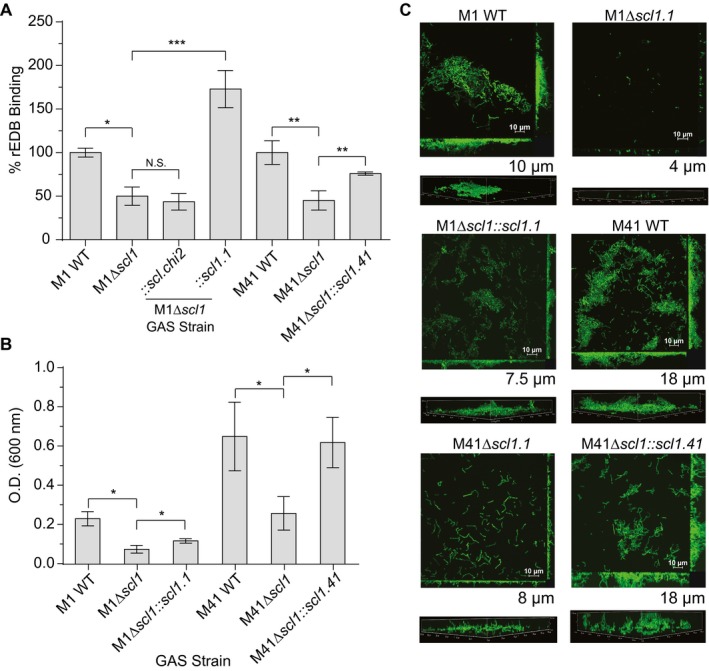
Scl1‐EDB binding mediates GAS adherence and biofilm formation. Binding of rEDB to whole GAS cells was compared between WT and Δs*cl1* mutants, as well as the contribution of surface Scl1 to GAS biofilm formation on rEDB‐coated surfaces. A. rEDB binding to whole GAS cells. Isogenic WT and *Δscl1* mutants of the M1‐ and M41‐type GAS strains were used, as well as the M1Δs*cl1* mutant complemented for the expression of native Scl1.1 (Δs*cl1::scl1.1*) or the chimeric Scl.chi2 (Δs*cl1::scl.chi2*) proteins, and M41Δs*cl1* mutant complemented for the expression of native Scl1.41 variant (Δs*cl1::scl1.41*). rEDB binding to whole GAS cells was detected by flow cytometry with primary anti‐His‐tag mAb; binding to GAS WT cells was set as 100%. Statistical analysis was calculated using Student's two‐tailed *t‐*test from three independent experiments (*N* = 3 ± SD); *P ≤ 0.05, **P ≤ 0.01, ***P ≤ 0.001. B. Assessment of biofilm formation on rEDB‐coated surfaces. M1 and M41 WT, Δs*cl1* isogenic mutants, and Δs*cl1* mutants complemented for the expression of native Scl1 variants were compared. Biofilm formation was evaluated spectrophotometrically following crystal violet staining. Graphic bars indicate the mean OD_600nm_ normalized against BSA controls. Statistical analysis was calculated using Student's two‐tailed *t*‐test from three independent experiments (*N* = 3 ± SD); *P ≤ 0.05. C. Microscopy imaging of GAS biofilms formed on rEDB coating. The same set of GFP‐expressing GAS strains shown in panel B were grown on rEDB‐coated glass coverslips for 24 h. Two‐dimensional orthogonal views of GAS biofilms are representative of Z stacks from 15 fields over two experiments. Average vertical thickness is indicated in micrometers below two‐dimensional orthogonal views, taken from 15 arbitrary fields over two experiments. [Colour figure can be viewed at wileyonlinelibrary.com]

First, we measured rEDB binding to whole GAS M1 and M41 WT cells, as compared to that for their corresponding isogenic Scl1*‐*deficient mutant cells, M1Δs*cl1* and M41Δs*cl1*. Levels of rEDB deposition onto GAS strains were determined by flow cytometry after a 30‐minute incubation (Fig. 2A). There was a significant reduction in rEDB deposition on the surface of M1Δs*cl1*, by ~50%, and M41*Δscl1*, by ~60%, compared to their respective WT strains, set as 100% binding‐level. We also tested rEDB binding to the M1Δs*cl1* mutant cells complemented *in trans* for the expression of either the original Scl1.1 protein (Δs*cl1::scl1.1*) or the chimeric Scl.chi2 variant (Δs*cl1::scl.chi2*), as well as the M41Δs*cl1* mutant complemented *in trans* to express the original Scl1.41 variant (Δs*cl1::scl1.41*). Restoration of expression of the rEDB‐binding‐positive protein Scl1.1 in the M1Δs*cl1* mutant restored rEDB‐binding, while M1Δs*cl1* mutant cells complemented for the expression of the rEDB‐binding‐negative protein Scl.chi2 did not show increased rEDB deposition. Similarly, complementation of the M41Δs*cl1* mutant for Scl1.41 expression restored WT rEDB‐binding phenotype. Residual rEDB detected on the surface of both Δs*cl1* mutants may reflect a non‐specific rEDB‐binding background in this assay, or the presence of an unknown GAS surface protein with capacity to bind rEDB. Collectively, our results indicate that GAS binding to EDB is mediated by Scl1.

Scl1 promotes biofilm formation on wound‐associated EDA/cFn (Oliver‐Kozup *et al.*, 2013; Bachert *et al.*, 2016). To understand if the GAS‐EDB interaction facilitates GAS biofilm formation *in vitro*, we used rEDB‐coated surfaces and analyzed biofilm formation by GAS isogenic strains. Biomass increases were detected spectrophotometrically, using crystal violet staining, 24 h after inoculating the wells with WT strains, compared to amounts of biomass of the isogenic M1Δs*cl1* and M41Δ*scl1* mutants (Fig. 2B). Restoration of Scl1 expression via complementation (Δs*cl1::scl1.1 or scl1.41)* also resulted in a significant increase in biomass compared to their respective isogenic Δs*cl1* mutants (Fig. 2B), although, complementation of the M1Δs*cl1* mutant resulted in partial biofilm restoration. These results were further supported through confocal laser scanning microscopy, where M1 and M41 WT strains formed biofilm structures on rEDB, with an average thickness of approximately 10 and 18 µm respectively (Fig. 2C). M1Δ*scl1* and M41Δ*scl1* mutant strains formed significantly reduced structures, with an average thickness of approximately 4 and 8 µm respectively. A 60% decrease in M1 and a 55% decrease in M41 was calculated when comparing WT and Δ*scl1* mutants. Restoration of Scl1 expression on the surface of M1 and M41 Δs*cl1* mutants resulted in the restoration of GAS biofilm structures on rEDB, accompanied by the increases in biofilm thickness recorded for both M types, compared to their respective Δs*cl1* mutants. Together, our data validate GAS capacity for binding to the fibronectin type III repeat EDB to facilitate GAS biofilm formation.

### Scl1 binds TnC via the Scl1‐V domain

**Figure 3:**
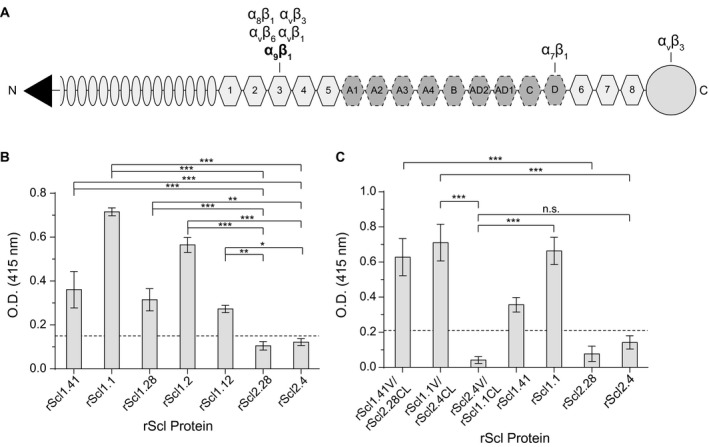
Characterization of rScl1 and rScl2 binding to tenascin‐C (TnC). For ligand binding by ELISA, rScl proteins were immobilized onto *Strep*‐Tactin‐coated microplate wells and incubated with full‐length TnC. Primary anti‐TnC mAb and HRP‐conjugated secondary Ab were used for ligand detection. Graph bars indicate the mean OD_415nm_ normalized against BSA controls. Statistical analysis was calculated using a one‐way ANOVA, from three independent experiments (unless noted otherwise), each performed in triplicate wells (*N* = 3 ± SD); *P ≤ 0.05, **P ≤ 0.01, ***P ≤ 0.001. Dashed line indicates threshold OD_415nm_ +2SD values recorded for binding‐negative rScl2.28 control protein. A. Schematic representation of full‐length TnC. Depicted are from the N‐terminus: assembly domain (*triangle*), epidermal growth factor‐like repeats (*ovals*), constitutively expressed fibronectin type III repeats 1‐5 and 6‐8 (*light hexagons*), alternatively spliced fibronectin type III repeats (*dark hexagons*), and fibrinogen‐related domain (*circle*). Known integrin‐binding domains are marked above the model. B. TnC binding to recombinant Scl1‐ and Scl2 ‐derived constructs. rScl1 and rScl2 panel represents diverse Scl1 and Scl2 variants originating from strains of diverse M types. Statistical significance evaluates the differences in TnC binding by rScl1 proteins from M41, M1, M28, M2 and M12 strains, as compared to rScl2 control proteins from M28 and M4 strains. C. Identification of the Scl1 domain responsible for TnC binding. A set of rScl proteins were tested for binding to TnC by ELISA that included the original rScl1 (rScl1.41, rScl.1) and rScl2 (rScl2.28, rScl2.4) proteins, as well as constructs generated via domain swapping; domain compositions for those rScl constructs are shown underneath the graph. Statistical significance evaluates the differences in TnC binding, as depicted on the graph. Two independent experiments were performed, using triplicate wells.

TnC is a multi‐domain glycoprotein present in wounded tissue with a central region consisting of up to 15 fibronectin type III repeats (Fig. 3A) (Sahlberg *et al.*, 2001; Midwood and Orend, 2009; Karus *et al.*, 2011; Chiquet‐Ehrismann *et al.*, 2014). We hypothesized that Scl1 could also bind to TnC through recognition of the FnIII repeats. We first investigated full‐length TnC binding using the same panel of rScl1 and rScl2 proteins used to investigate rEDB‐binding. TnC binds to all Scl1‐derived constructs (Fig. 3B). In contrast, Scl2‐derived constructs demonstrate significantly lower levels of binding and exhibited little to no binding, reminiscent of their lack of binding to cFn, rEDA and rEDB. To determine if Scl1 binding to TnC was mediated through the Scl1‐V domain, we utilized rScl constructs generated via domain replacement, derived from the ECM‐binding‐positive constructs rScl1.1 and rScl1.41 and the binding‐negative constructs rScl2.4 and rScl2.28. Both Scl2‐based constructs, still harboring the original Scl2‐CL domains that acquired Scl1‐V domains (rScl1.41V/Scl2.28CL and rScl1.1V/Scl2.4CL) also acquired the capacities of TnC binding, compared to the parental proteins rScl2.28 and rScl2.4. Conversely, substitution of the V‐domain in binding‐positive rScl1.1 with the binding‐negative rScl2.4 V‐domain (rScl2.4V/rScl1.1CL) eliminated TnC binding. These data demonstrate that TnC binding is exclusive to Scl1 and is mediated by the globular V‐domain, a mechanism conserved among diverse Scl1 variants, underscoring the importance of the Scl1‐TnC interaction in GAS wound colonization.

### Scl1 binds to the type III repeats of TnC  via surface exposed loops of the V‐domain

**Figure 4:**
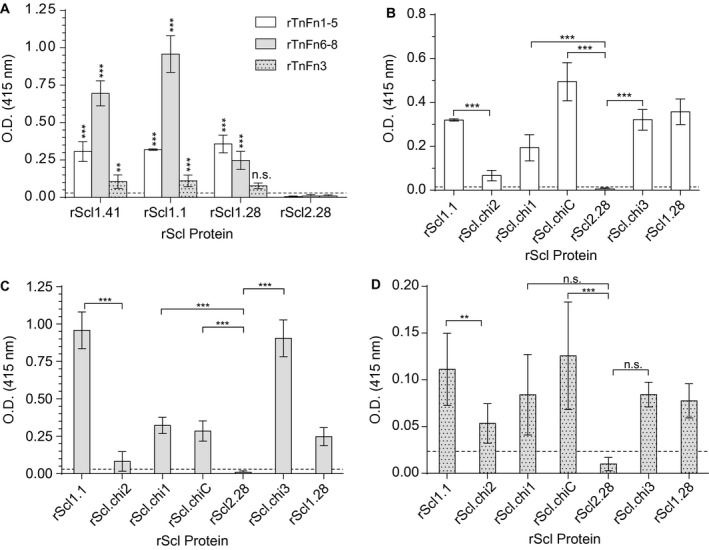
Characterization of rScl1 binding to recombinant fibronectin type III repeats in tenascin‐C (rTnFnIII). For ligand binding by ELISA, rScl proteins were immobilized onto *Strep*‐Tactin‐coated microplate wells and incubated with recombinant TnC fragments comprising of rTnFnIII repeats. Primary anti‐rTnFnIII (anti‐His‐tag) mAb and HRP‐conjugated secondary Ab were used for ligand detection. Graph bars indicate the mean OD_415nm_ normalized against BSA controls. Statistical analysis was calculated using a one‐way ANOVA from three independent experiments, each performed in triplicate wells (*N* = 3 ± SD); **P ≤ 0.01, ***P ≤ 0.001. Dashed line indicates threshold OD_415nm_ +2SD values recorded for binding‐negative rScl2.28 control protein. A. Binding of rTnFnIII to original rScl1 and rScl2 proteins. Statistical significance evaluates the difference in rTnFnIII binding by rScl1 proteins, as compared to TnC‐binding‐negative rScl2.28 control protein. B–D. Binding of rTnFnIII constructs to original and chimeric rScl proteins. rScl binding by rTnFn1‐5 (B), rTnFn6‐8 (C) and rTnFn3 (D) is shown.

Within EDA, Scl1 binds at or near the α_9_β_1_‐integrin‐binding site (Oliver‐Kozup *et al.*, 2013), an essential receptor on human cells (Shinde *et al.*, 2008), which also binds to the third FnIII repeat in TnC (TnFn3) (Yokosaki *et al.*, 1998) (Fig. 3A). Therefore, we hypothesized that Scl1 may specifically recognize and bind to a yet unknown sequence on the TnFn3 repeat within the TnC molecule. To test this, we produced recombinant fragments, representing the constitutively expressed TnFnIII regions encompassing repeats 1‐5 (rTnFn1‐5), repeat number 3 (rTnFn3) and repeats 6‐8 (rTnFn6‐8); the latter construct to be used as a binding‐negative control. We first tested the rTnFnIII constructs for binding to TnC‐binding positive rScl1.1, rScl1.28 and rScl1.41 proteins and a control TnC‐binding‐negative rScl2.28 construct (Fig. 4A). Binding of the rTnFn1‐5 fragment to rScl1 constructs was significantly higher than to the rScl2.28 control. However, rTnFn3 binding to rScl1.1 and rScl1.41 constructs was greatly reduced, although statistically significant, compared to rScl2.28 binding. Unexpectedly, we detected substantial rTnFn6‐8 binding to rScl1 proteins, significant compared to rScl2.28 control. These data indicate that rScl1 proteins bind to the constitutively expressed TnFnIII repeats 1‐5 and 6‐8 of TnC but not to, the single rTnFn3 construct.

Since the Scl1‐V domain binds TnC (Fig. 3C) via type III repeats (Fig. 4A), we tested the involvement of the V‐domain loop‐segment in the recognition of the TnFnIII repeats. We employed the same set of rScl proteins that were used earlier in the analysis of rScl1‐V interactions with rEDB (Fig. 1C) to evaluate binding by rTnFn1‐5 (Fig. 4B), rTnFn6‐8 (Fig. 4C) and rTnFn3 (Fig. 4D). The chimeric proteins rScl.chi1, rScl.chiC and rScl.chi3, positive for rEDA and rEDB binding, also demonstrated significant binding to rTnFn1‐5 and rTnFn6‐8, compared to rScl2.28 control. The chimeric construct rScl.chi2 showed a significant reduction in binding to both rTnFn1‐5 and rTnFn6‐8. rTnFn3 bound to rScl1 constructs at low levels and detection was significant only for rScl.chiC, but not rScl.chi1 nor rScl.chi3, compared to rScl2.28 control. In addition, there was a significant reduction of rTnFn3 binding to rScl.chi2, compared to rScl1.1‐loop recipient, together, indicating that the limited binding by rTnFn3 is mediated by the loop‐region. In summary, we have demonstrated for the first‐time direct binding between rScl1 and rTnFnIII fragments that is mediated by Scl1‐V loop‐region, essential for recognition of multiple FnIII repeats in cFn and TnC.

### Scl1 supports GAS attachment to EDA‐, EDB‐ and TnC‐containing ECM deposited by cancer‐associated fibroblasts

**Figure 5:**
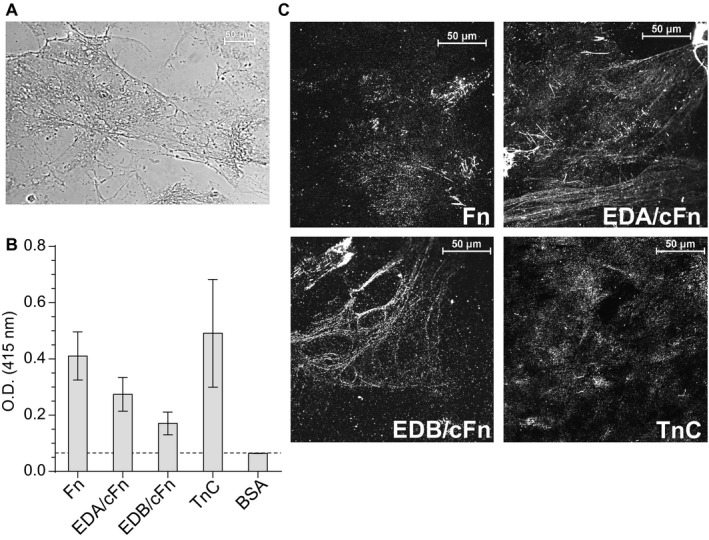
Characterization of extracellular matrices deposited by cancer‐associated fibroblasts (CAFs). CAFs were isolated from a stage IV laryngeal primary tumor and grown to confluency. Matrices were prepared after the removal of cells by treatment with EGTA and were then evaluated for the presence of EDA‐ and EDB‐containing fibronectins and TnC. A. Visualization of the overall structure of ECM deposited by CAFs. Ponceau S staining reveals complex fibrillary network of the matrices used in this study. B. Characterization of the ECM deposited by CAFs. The presence of total Fn, EDA/cFn, EDB/cFn and TnC was assessed by ELISA with specific mAbs and secondary HRP‐conjugated antibody. Graph bars indicate the mean OD_415nm_ from three independent experiments, each with triplicate wells (*N* = 3 ± SD). Dashed line indicates threshold OD_415nm_ +2SD values recorded for BSA control wells. C. Immunofluorescent visualization of the ECM deposited by CAFs. CAF‐deposited matrices prepared on glass coverslips were incubated with primary mAbs specific for Fn, EDA/cFn, EDB/cFn and TnC, followed by secondary Ab conjugated with Alexa Fluor® 568. Images were taken using confocal microscope with 60× objective; representative images are shown from two independent experiments, imaging 10 arbitrary fields per coverslip.

Solid tumors display similarities to healing wounds, albeit persistently, including extensive ECM deposition and remodeling, due in large part to stromal cancer‐associated fibroblasts (CAFs) (Gaggioli *et al.*, 2007; Schäfer and Werner, 2008; Marsh *et al.*, 2013; Dvorak, 2015; Gopal *et al.*, 2017). CAFs deposit EDA/EDB‐fibronectins and TnC (Mackie *et al.*, 1987; Norton and Hynes, 1987; Rybak *et al.*, 2007; O'Connell *et al.*, 2011; Marsh *et al.*, 2013). Therefore, we sought to investigate if Scl1 mediates GAS attachment to the ECM deposited by CAFs. First, we characterized our *in vitro* model for the composition of the extracellular matrix deposited by primary CAFs, isolated from a stage IV laryngeal tumor tissue. Matrices were initially visualized using Ponceau S staining, showing good overall integrity of a complex fibrillary network, without obvious signs of degradation (Fig. 5A). This matrix contained Fn, EDA/cFn, EDB/cFn and TnC, when evaluated by ELISA, using specific monoclonal antibodies for each ECM subtype (Fig. 5B). Immunofluorescence imaging confirmed the presence of cFn isoforms that contain EDA and EDB, as well as the presence of TnC within CAF‐deposited ECM (Fig. 5C). Specificity of each of the monoclonal antibodies, as well as background control images are shown in Fig. S1.

**Figure 6:**
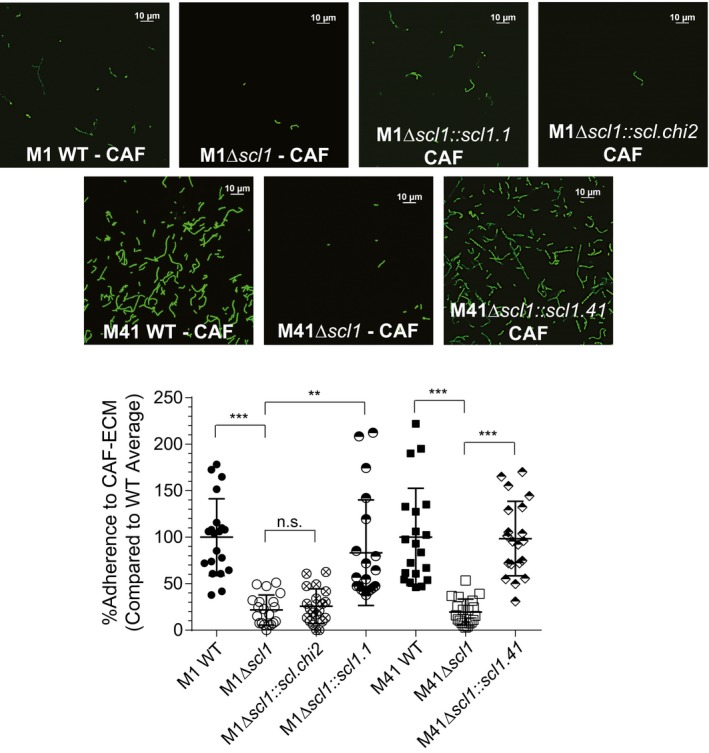
Scl1‐mediated GAS attachment to ECM‐deposited by cancer‐associated fibroblasts (CAFs). Isogenic WT M1 and M41 GAS strains, their Δ*scl1* mutants, and *trans‐*complemented strains to restore Scl1 expression in each mutant, or express Scl.chimera2, were compared for the attachment to CAF‐derived ECM. GFP‐GAS strains were inoculated onto CAF‐derived ECM coatings, allowed to attach for 1 h, and imaged using fluorescent confocal microscope with 100x objective. *Top*, representative images of attached strains are shown. *Bottom*, quantification of GAS attachment. Bacteria were counted in 20 random fields, and the average from all 20 fields was calculated with WT binding set as a 100%. Statistical significance was calculated using a one‐way ANOVA from three independent experiments, each performed in duplicate wells (*N* = 3 ± SD); **P ≤ 0.01, ***P ≤ 0.001. Statistical analysis evaluates the difference between adherence to CAF‐derived matrices by the WT and their respective isogenic Δs*cl1* mutants. Each symbol shown represents one imaged‐field. [Colour figure can be viewed at wileyonlinelibrary.com]

We next used the matrices deposited by CAFs as a substratum for studying the Scl1‐mediated GAS attachment. The GFP‐expressing M1 and M41 WT strains, their isogenic Δ*scl1* mutants and *trans*‐complemented mutant cells, used in flow cytometry experiments in Fig. 2, were compared for the adherence to glass coverslips containing CAF‐deposited matrices (Fig. 6). More fluorescent M1 and M41 WT GAS were seen adhered to the CAF‐derived ECM on coverslips than their respective M1Δ*scl1* and M41Δs*cl1* mutants (Fig. 6, *top*). Complementation of Scl1 expression in both Δ*scl1* mutants resulted in increased adherence to CAF‐ECM comparable to their respective WT strains. Additionally, expression of Scl.chi2, harboring inactive Scl2‐loop, by M1Δ*scl1*, did not reveal enhanced adherence. Quantification of the number of attached cells show that significantly fewer, by approximately 70%, of the M1Δ*scl1* cells bound the matrices compared to the WT strain. Similarly, the M41Δs*cl1* mutant bound the matrices ~80% less than the WT strain. Restoration of Scl1.1, but not Scl.chi2, and Scl1.41 expression on Δscl1 mutants rescued the respective WT phenotypes (Fig. 6, *bottom*). Therefore, our results show that Scl1 mediates GAS adherence to the ECM deposited by CAFs, and this adherence is mediated by the Scl1‐V domain loop‐region.

**Figure 7:**
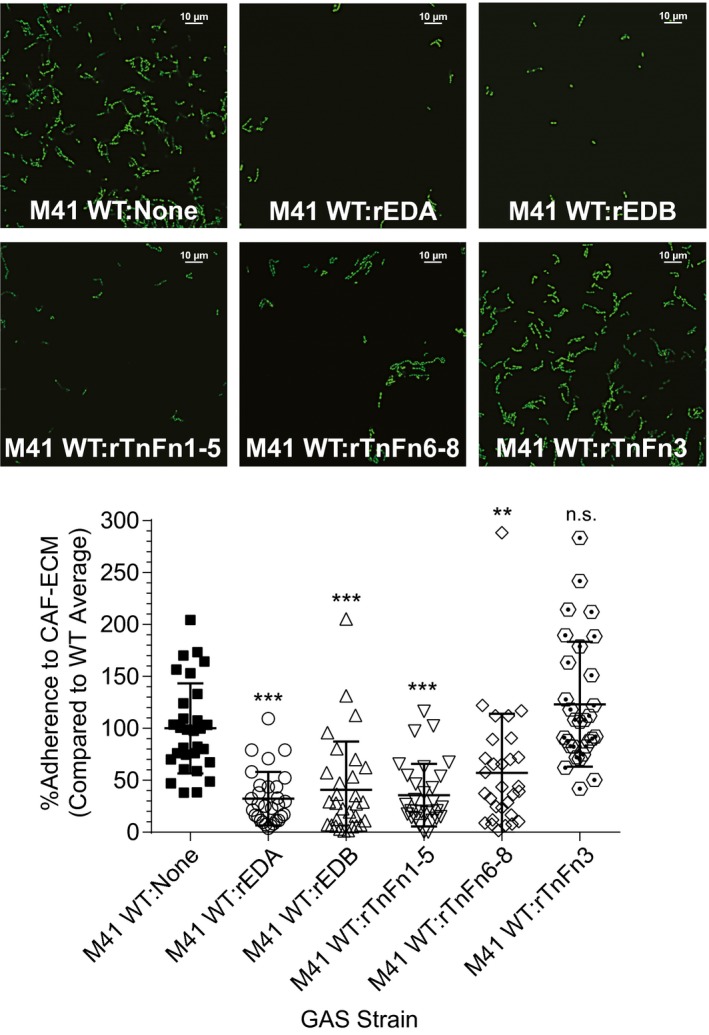
Specificity of Scl1‐mediated GAS attachment to cancer‐associated fibroblast matrices. WT GFP‐M41 strain was pre‐incubated with recombinant ECM ligands for 30 min, prior to attachment assay on CAF‐derived ECM. Recombinant ECM constructs included rEDA, rEDB, rTnFn1‐5, rTnFn6‐8 and rTnFn3. GAS were allowed to attach for 1 h and then imaged using fluorescent confocal microscope with 100× objective. *Top*, representative images and *Bottom*, quantification of GAS attachment with WT binding set as 100%. Bacteria were counted in 30 random fields, and the average from all 30 fields was calculated. Percentage based off of the average number of counted bacteria for the parental WT strain. Statistical significance was calculated using a one‐way ANOVA from two independent experiments, each performed in duplicate wells (*N* = 3 ± SD); **P ≤ 0.01, ***P ≤ 0.001. Statistical analysis evaluates the difference in adherence between WT GAS and WT GAS pre‐incubated with rECM competitor. Each symbol represents one imaged‐field. [Colour figure can be viewed at wileyonlinelibrary.com]

In a follow‐up attachment inhibition experiment, we tested if Scl1 specifically mediates GAS adherence by binding to EDA/ and EDB/ cFn isoforms, as well as to the TnFnIII repeats within CAF‐derived ECM. M41 WT GAS was used since this strain showed better 1‐h attachment levels to CAF‐ECM, compared to the M1‐type strain (Fig. 6). GAS cells were pre‐incubated with rEDA, rEDB, rTnFn1‐5, rTnFn6‐8 or rTnFn3 for 30 min prior to a 1‐h incubation of GAS on CAF‐derived ECMs (Fig. 7). Pre‐incubation of GAS with rEDA and rEDB resulted in a ~70% and ~60% reduction in the number of adherent cells, respectively, compared to untreated WT control. This level in attachment inhibition of the WT strain by rFnIII constructs results in a decreased level of attachment, similar to levels reported for the Δ*scl1.41* mutant in Fig. 6. Similarly, pre‐incubation with rTnFn1‐5 and rTnFn6‐8 resulted in a ~70% and ~40% reduction in GAS adherence to the CAF‐derived matrices, while GAS pre‐incubated with rTnFn3 showed no differences in attachment compared to untreated WT control (Fig. 7). These data demonstrate that Scl1 specifically mediates GAS adherence to cFn isoforms that contain EDA/ and EDB/cFn, as well as to TnFnIII repeats, within the complex ECM deposited by CAFs. CAF‐deposited ECM also supports Scl1‐mediated GAS biofilm formation, beyond the initial attachment, using crystal violet staining and confocal fluorescence microscopy (Fig. S2).

To assess the role of Scl1 in mediating GAS binding to ECM produced by cancer cells, we applied the same *in vitro* analysis using the Saos‐2 osteosarcoma cell line (Fogh *et al.*, 1977). This line produced an ECM structure, stained with Ponceau S, containing EDA/ EDB cFn isoforms and TnC (Fig. S3AB). Saos‐2 ECM supported the attachment of M1 and M41 WT GAS, while M1Δs*cl1* and M41Δs*cl1* mutants demonstrated significantly reduced binding (Fig. S3C). Crystal violet staining after 24 h shows significantly larger bacterial biomass grown by both WT strains on Saos‐2‐derived ECM, compared with their respective Δs*cl1* mutants (Fig. S3D). Therefore, our results show that Scl1 also mediates GAS adherence and biofilm formation to the ECM deposited by Saos‐2 cells.

## Discussion

**Figure 8:**
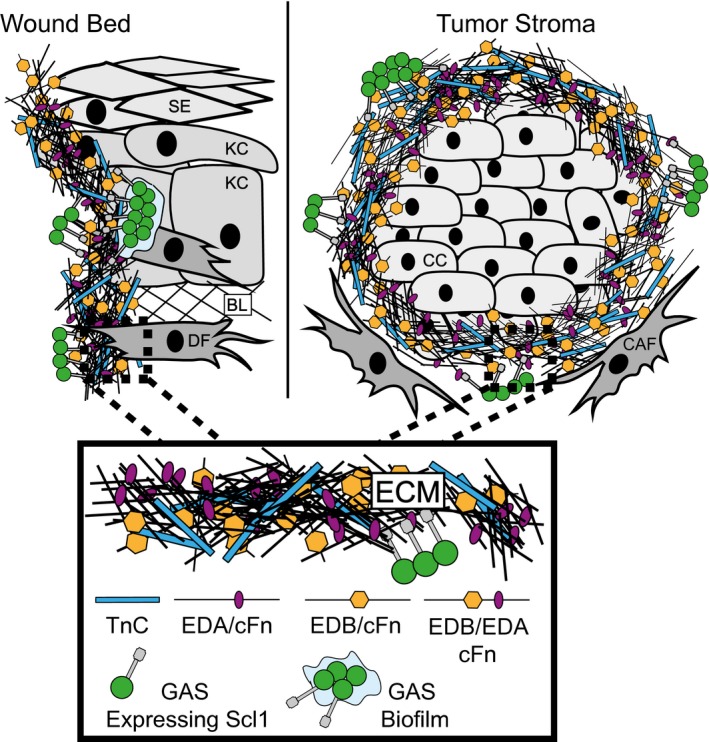
Model of GAS colonization of wound and tumor microenvironments. The wound and tumor microenvironments are enriched in isoforms of cellular fibronectin (cFn) that contain extra domain A (EDA) and extra domain B (EDB), as well as tenascin‐C (TnC). *Left*, GAS gains access to the host via portal of entry, such as through a breach in keratinized squamous epithelium (SE), into a tissue environment that contains keratinocytes (KC), basal lamina (BL) ECM and dermal fibroblasts (DF). Within wound, cells such as DFs deposit cFn isoforms that contain EDA and EDB, as well as TnC. GAS‐Scl1 adhesin binds EDA and EDB of cFn, and TnC, promoting call attachment and tissue microcolony formation within the wound. *Right*, Cancer cells (CC) are surrounded by cancer‐associated fibroblasts (CAFs), which deposit cFn isoforms that contain EDA and/or EDB, and TnC, recognized by GAS‐Scl1. *Enlarged insert*, close‐up view of the wound‐ and tumor‐associated ECM. [Colour figure can be viewed at wileyonlinelibrary.com]

GAS infections start within the wounded portal of entry, which is characterized by a microenvironment rich in cFn isoforms that contain either EDA and/or EDB, as well as TnC. Here, we report that Scl1 of GAS binds to those multiple wound‐associated ECM targets that contain FnIII‐type repeats (Fig. 8). Binding to FnIII ligands was determined in different Scl1 variants by the same conserved structural element within the Scl1‐V domain and facilitated GAS adherence and biofilm formation.

Group A streptococcal strains may express numerous Fn‐binding proteins on the cell surface and some 11 distinct Fn adhesins have been reported (reviewed in (Yamaguchi *et al.*, 2013). The most common mechanism of Fn binding is via the classical Fn‐binding repeats which are found in several Fn‐binding proteins of GAS, such as protein F1/SfbI and F2/PFBP, serum opacity factor SOF/SfbII, FbaA and FbaB, SfbX and Fbp54 (Signäs *et al.*, 1989; Talay *et al.*, 1992; Rakonjac *et al.*, 1995; Jaffe *et al.*, 1996; Terao *et al.*, 2001; Schwarz‐Linek *et al.*, 2003). During the binding process, the Fn‐binding repeats of those adhesins, interact with the N‐terminal FnI repeats through a tandem β‐zipper mechanism and contribute an additional anti‐parallel β‐strand within the β‐sheet structure (House‐Pompeo *et al.*, 1996; Schwarz‐Linek *et al.*, 2003). Scl1, however, lacks the classical Fn‐binding repeats and binds instead to the FnIII repeat EDA, via its globular V‐domain (Oliver‐Kozup *et al.*, 2013). The Scl1‐EDA binding mechanism is mediated by the C‐C′ loop of EDA and surface‐exposed loops of the Scl1‐V domain (McNitt *et al.*, 2018). Here, we determined that several rScl1 variants, with diverse V‐domain sequences, bind to rEDB polypeptide. To our knowledge, Scl1 is the first bacterial adhesin that directly binds the FnIII‐EDB of cFn. Furthermore, using a series of engineered rScl constructs, we show that Scl1‐EDB binding engages, like Scl1‐EDA binding, the Scl1‐V‐domain loops and suggests the same conserved binding mechanism. Homology modeling of EDB and analysis of the predicted loop sequences imply that the C‐C′ loop of EDB is a potential Scl1‐binding target, which requires future experimental support.

Here, we show that the Scl1‐EDB interaction facilitates GAS biofilm formation *in vitro* on rEDB‐coated surfaces. The role of EDB in bacterial infections is beginning to be elucidated. Recently, cFns that include the EDB segment were shown to be upregulated in the cerebral spinal fluid and serum of patients suffering from *Staphylococcus aureus* meningitis (Kraft *et al.*, 2016). EDB‐containing cFns in these patients were released by immune cells during phagocytosis of *S. aureus*, accompanied by bacterial removal via phagocytic cells. Phagocytosis of *S. aureus in vitro* was augmented by the addition of exogenous EDB/cFn, which activated the α_v_β_3_ integrin on phagocytic cells. However, it was not shown if *S. aureus* adhered directly to EDB (Kraft *et al.*, 2016).

Two streptococcal proteins have been reported to bind to TnFnIII: protein H (Frick *et al.*, 1995) and protein F2 (Herrera *et al.*, 2018). The M‐like protein H binds factor H and IgG, as well as the neural cell adhesion molecule (N‐CAM), which contains FnIII repeats (Hemperly *et al.*, 1986). Protein H binding to N‐CAM was competitively inhibited with pFn fragments, encompassing the FnIII repeat region, as well as with two additional FnIII‐containing recombinant proteins derived from TnC (Frick *et al.*, 1995). Protein F2 contains the prototypical fibronectin‐binding repeats that bind to FnI repeats (Jaffe *et al.*, 1996; Kreikemeyer *et al.*, 2004). To our knowledge, direct binding to the FnIII repeats in either fibronectin or TnC by F2 protein has not been reported. A recent study showed that a protein F2‐deficient mutant of an M3‐GAS strain bound significantly less TnC compared with the wild‐type parental organism, which was restored by *in*‐*trans* complementation (Herrera *et al.*, 2018). It should be noted that the M3 strain used in this study, MGAS315, does not expresses the Scl1 adhesin (Bachert *et al.*, 2016). Here, we used defined recombinant TnFnIII fragments, rTnFn1‐5 and rTnFn6‐8, and show for the first time that Scl1 binds directly to the constitutively expressed FnIII repeats of TnC, implicating Scl1 in binding to all TnC isoforms overexpressed in the wound.

EDA/EDB/‐containing cFn isoforms and TnC are also associated with solid tumors and metastatic cancers, wherein resident cancer cells activate the surrounding stromal fibroblasts, known as cancer‐associated fibroblasts (CAFs) (Erez *et al.*, 2010; Kalluri, 2016; Gopal *et al.*, 2017). While both normal fibroblast and CAFs secrete and deposit cFns (Hynes, 1990), as we show here cultured CAFs, isolated from a stage IV laryngeal primary tumor, deposit cFn isoforms as well as TnC *in vitro*. Moreover, we report that Scl1 facilitates GAS adherence to and biofilm formation on CAF‐deposited ECM preparations by specific binding to EDA, EDB and the TnFnIII repeats.

These data enable comparison to an intriguing historical connection to cancer therapy, in which GAS was an original and major component of Coley's toxin (succinctly reviewed in [McCarthy, 2006]). Developed by Dr. William Coley, a bone sarcoma surgeon (1862–1936), Coley's toxin was the first use of a cancer immunotherapy in medicine (Burdick, 1937; McCarthy, 2006). Coley and colleagues injected live and killed streptococci to treat patients afflicted with soft tissue and bone tumors with relative success (Decker *et al.*, 2017). Interestingly, we now know that osteosarcoma cells express cFn isoforms that contain EDA and/or EDB, as well as TnC (Kilian *et al.*, 2004; 2008; Sun *et al.*, 2018). Here we used the osteosarcoma Saos‐2 cells to produce model matrices, containing those FnIII targets and showed that Scl1 promotes GAS colonization and biofilm formation on Saos‐2‐derived matrices. Our work provides a previously unrealized opportunity in the utilization of GAS in the potential treatment of cancer. Discerning the capacity for GAS to form biofilms within CAF‐ and Saos‐2‐derived ECM, and the role of Scl1 in this process, is important since GAS has been under pre‐clinical (Linnebacher *et al.*, 2008; Maletzki *et al.*, 2008) and clinical (Yamaguchi *et al.*, 2003; Roh and Park, 2008; Nohara *et al.*, 2016; Wang *et al.*, 2019) evaluation as a cancer therapeutic.

Understanding of the GAS‐fibronectin interaction has evolved since our finding that the GAS adhesin, Scl1, selectively binds to cFn, but not pFn, (Caswell *et al.*, 2010) via a unique mechanism involving the cFn type III repeat, EDA (Oliver‐Kozup *et al.*, 2013; McNitt *et al.*, 2018). Here, we demonstrate that Scl1 also binds to EDB in isoforms of cFn, as well as to the FnIII repeats within TnC. All three ECM ligands are expressed within wounded tissue, which is typically the first site GAS encounters within the host. In each case, surface‐exposed loops of the globular sequence‐variable, but structurally conserved, V‐domain of Scl1 were responsible for ligand recognition and binding. The Scl1 adhesin improves GAS adherence and biofilm formation on extracellular matrices rich in cFn isoforms that contain EDA and/or EDB, and TnC. Interestingly, the aforementioned ECM components are also constituents of tumor microenvironment and our initial experiments suggest Scl1 has the capacity for targeting solid tumors. In summary, the Scl1‐V domain has evolved within the wound microenvironment, driven by selection for binding to multiple ECM components containing wound‐associated FnIII repeats.

## Experimental procedures

### Bacterial strains and growth

Group A *Streptococcus* (GAS) strains MGAS5005‐M1 and MGAS6183‐M41, as well as their *scl1*‐inactivated isogenic mutants were used in this study (Hoe *et al.*, 1999; Lukomski *et al.*, 2000; Han *et al.*, 2006a; Caswell *et al.*, 2007). Briefly, both mutants were generated by allelic replacement with nonpolar resistance cassettes, encoding spectinomycin (MGAS5005 Δ*scl1*) and erythromycin (MGAS6183 Δ*scl1*) resistance. GAS cultures were grown at 37°C, with 5% of CO_2_ in Todd‐Hewitt broth supplemented with 0.2% of yeast extract and on Brain Heart Infusion (BHI) agar. For GAS antibiotic selection, erythromycin (4 µg ml^−1^), chloramphenicol (5–10 µg ml^−1^) and spectinomycin (100 µg ml^−1^) were added to the medium.

### Complementation of M1 and M41 group A *Streptococcus*

To complement MGAS5005 Δ*scl1* with either *scl1.1* or *scl.chi2 in trans*, the *E.coli* shuttle vector pSB207 was used (Cramer *et al.*, 2003). Briefly, a DNA fragment, encompassing the *scl1.1* coding sequence with upstream promoter, was PCR‐amplified from genomic DNA and cloned into pSB207, generating plasmid pSL620 (McNitt *et al.*, 2018). A synthetic double‐stranded DNA fragment (gBlocks; Integrated DNA Technologies) encoding the *scl.chi2* coding sequence was cloned into pSL620, generating pSL621 (McNitt *et al.*, 2018). Clones were verified by sequencing and were then introduced into MGAS5005 Δ*scl1* mutant; transformants were selected on BHI agar containing 10 µg ml^−1^ of chloramphenicol and mutant cultures were routinely grown in THY broth with 10 µg ml^−1^ of chloramphenicol. Complementation of MGAS6183 Δ*scl1* with *scl1.41* was done as previously described (Caswell *et al.*, 2007). Briefly, the *scl1.41* allele and promoter sequence were cloned into shuttle vector pJRS525 (McIver and Scott, 1997), and introduced into MGAS6183. Transformants were grown in THY broth with 50 µg ml^−1^ of spectinomycin.

### Recombinant protein production

#### Production of recombinant streptococcal collagen‐like proteins (rScls)

All rScl proteins were generated using the *Strep*‐tag II cloning, expression and purification system (IBA‐GmbH). Gene cloning and rScl‐protein production were performed in *E. coli* DH5a, TB1 and BL‐21 strains grown in Luria‐Bertani (LB) media with ampicillin (100 µg ml^−1^) at 37°C. rScl‐encoding clones, which were derived from the original *scl* alleles, were generated by PCR amplification from GAS genomic DNA and cloned into the *E.coli* expression vector pASK‐IBA2 (Xu *et al.*, 2002; Han *et al.*, 2006b). Clones encoding the chimeric rScl proteins were generated using synthetic double‐stranded DNA fragments (gBlocks; Integrated DNA Technologies), as described previously (McNitt *et al.*, 2018). All plasmids were verified by DNA sequencing. Domain swapped rScl proteins were generated as described previously (Caswell *et al.*, 2010).

Proteins were expressed with a C‐terminal affinity tag and purified on *Strep*‐Tactin sepharose, as described (Xu *et al.*, 2002; Han *et al.*, 2006b). The rScl1.1 protein is derived from Scl1 protein in M1‐type strain MGAS6708 (Xu *et al.*, 2002); rScl1.28 originates from M28 strain MGAS6274 (Xu *et al.*, 2002), rScl1.41 is derived from M41 strain MGAS6183 (Humtsoe *et al.*, 2005), rScl1.3 is derived from M3 strain MGAS315 (Bachert *et al.*, 2016), rScl1.12 is derived from an M12 strain MGAS6139 (Han, Zwiefka *et al.*, 2006b), rScl1.2 is derived from an M2 strain MGAS3803 (Caswell *et al.*, 2008), and rScl2.4 is derived from M4 strain MGAS321 (Han, Zwiefka *et al.*, 2006b). Both naturally derived and chimeric rScl proteins were expressed in *E. coli* BL21 periplasm following induction with anhydrotetracycline at 0.2 µg ml^−1^ for 3 h. Cells were centrifuged and suspended either in high sucrose buffer (100 mM of Tris‐HCl, 1 mM of EDTA, pH 8.0, 500 mM of sucrose) or Cell Lytic B Buffer (Sigma), for separation of the periplasmic fraction and subsequent affinity purification. Purified proteins were analyzed by SDS‐PAGE and stained with RAPID*stain* ^TM^; proteins were dialyzed against 25 mM of HEPES, pH 8.0 and stored at −20°C. Protein concentrations were determined using Qubit fluorometric quantitation.

#### Recombinant extra domain B (rEDB) production

rEDB was produced using the pQE‐30 His‐tag cloning, expression, and purification system in the *E. coli* strain JM‐109, as described elsewhere (Kelsh *et al.*, 2014). EDB‐encoding segment was amplified by PCR from rat cDNA and cloned into pQE‐30; the resulting construct was a gift from Dr. John Peters. Protein expression was induced with 1 mM of isopropyl β‐d‐1‐thiogalactopyranoside for 3 h. Cells were harvested by centrifugation, and pellets were frozen at −20°C for 2 h or overnight. Cells were next suspended in a bacterial lysis buffer (50 mM of Tris/HCL pH 8.0, 50 mM of NaCl, 2 mM of MgCl_2_, 2% of Triton X‐100, 10 mM of β‐mercaptoethanol, 0.2 mg/ml of lysozyme, 1 EDTA‐free protease inhibitor cocktail tablet [per 10 ml], 1 mM of phenylmethane sulfonyl fluoride, 10 U ml of DNaseI) and incubated on ice for 20 min. Cell lysate was centrifuged at high speed (16,000 *g* × 20 min) and supernatant was collected. Supernatant was mixed 1:1 volumetrically with wash buffer (50 mM of NaH_2_PO_4_, 10 mM of imidazole and 300 mM of NaCl), added to 1 ml of cobalt‐agarose resin and then poured into a column. Sample was washed with 10× resin bed volume of wash buffer, and then rEDB protein was eluted in elution buffer (50 mM of NaH_2_PO_4_, 150 mM of imidazole and 300 mM of NaCl). Purified protein was dialyzed against 25 mM of HEPES buffer, pH 8.0 and stored at −20°C until future use. Protein integrity and purity were assessed by 18% of SDS‐PAGE and concentration was measured with Qubit fluorometric quantitation.

#### Production of recombinant fibronectin type III fragments of tenascin‐C (rTnFnIII)

Recombinant tenascin‐C fibronectin type III (FnIII) fragments: repeats 1‐5, (rTnFn1‐5), repeats 6‐8, (rTnFn6‐8) or repeat 3 (rTnFn3) were produced after recloning into pQE‐30 His‐tag vector in the *E. coli* strain XL1‐blue. Sequences for each fragment were PCR‐amplified from the original constructs cloned in pET15b (rTnFn1‐5 and rTnFn6‐8) or pET11b (rTnFn3) expression vectors (provided by Dr. Harold P. Erickson)(Aukhil *et al.*, 1993). Recombinant proteins were expressed, purified and stored as described above with rEDB.

#### Homology modeling of Scl chimeras

Three‐dimensional models of all variants of Scl proteins were constructed using the I‐TASSER protein modeling server. The crystal structure of Scl2.3 (PDB code 4nsm) (Squeglia *et al.*, 2014), which shares sequence identities in the range 30–40% with the Scl variants to model (Table S1), was used as a template out of the 10 top templates chosen from the LOMETS threading program. The I‐TASSER server builds models through an exhaustive process involving automatic template selection, energy evaluation and optimization of the hydrogen‐bonding network (Yang *et al.*, 2014). Visualization and analysis of the obtained models were performed using PyMOL (Roy *et al.*, 2010). R.m.s.d. deviations from the crystal structure of Scl2.3 domain and structure superpositions were performed using DALI (Laakso and Holm, 2016). For modeling of the CL region of the entire Scl structures, the crystal structure of the collagen triple helix [(PPG)_10_]_3_ (PDB code 1k6f) (Berisio *et al.*, 2002), was used as a template.

#### Protein binding assays

rScl proteins (0.5 μM of solutions) were immobilized onto *Strep*‐Tactin‐coated microplate wells for 1.5 h at room temperature and blocked with 1xTBS (25 mM of Tris, 150 mM of sodium chloride, pH 7.4) supplemented with 1% of fetal bovine serum albumin overnight at 4°C, followed by incubation with ECM ligands: rEDB, tenascin‐C (TnC) purified from glioblastoma cells (Sigma), rTnFn1‐5, rTnFn6‐8 and rTnFn3. The no rScl controls were performed in BSA‐coated wells for each ligand and each antibody used. Final OD values were normalized by subtracting the BSA controls in each experimental set‐up. ECM ligands were added to the rScl‐immobilized wells at 1 µg per well (except for TnC, incubated at 0.5 µg per well) and incubated at room temperature for 1 h. Bound rECM ligands were detected with monoclonal antibodies (mAbs): anti‐His‐tag for rEDA, rEDB, rTnFn1‐5, 6‐8 and 3 (Proteintech; 1:1,000), anti‐TnC BC‐24, specific for the epidermal growth factor‐like repeats, (ThermoFisher; 1:1,000), followed by goat anti‐mouse secondary antibody conjugated to horseradish peroxidase (HRP) (Jackson ImmunoResearch; 1:1,000). The HRP substrate, 2,20‐azino‐bis(3‐ethylbenzthiazoline‐6‐sulfonic acid) (ThermoFisher; ABTS) was used and colorimetric reactions were recorded at OD_415 nm_.

For concentration‐dependent binding, rScl proteins (0.25 µM solutions) were immobilized onto *Strep*‐Tactin‐coated microplate wells and incubated with increasing concentrations (0.1, 0.25, 0.5, 1.0 and 2.5 µM) of rEDB for 1 h, and processed as described above.

### Eukaryotic cell assays

#### Isolation of cancer‐associated fibroblasts (CAFs)

CAFs were isolated from a stage IV laryngeal cancer resection obtained from the West Virginia University Pathology Laboratory for Translational Medicine in compliance with approved Institutional Review Board protocol #1310105737A033 as described previously (Kumar *et al.*, 2018). Briefly, tissue was submerged in DMEM/10% FBS containing 1% of penicillin‐streptomycin‐amphotericin B (P‐S‐A) (Millipore, 51610420ML) and mechanically digested. Tissue pieces <2 mm were placed into a 24‐well plate, allowed to adhere for 2–3 min, then covered with media and placed in a humidified 37°C incubator with 5% of CO_2_. Emanating CAFs were cultured in DMEM supplemented with 10% of FBS/1% of P‐S‐A, passaged 2–3 times with Accumax (Millipore, SCR006) until acclimated, then passaged with 0.25% of trypsin and grown in DMEM/10% of FBS. CAFs were passaged five times before being cryopreserved. Thawed CAFs were passaged two times prior to use.

#### Preparation of cancer cell‐derived extracellular matrices

Cancer‐associated fibroblasts (CAFs) and the osteosarcoma cell line Saos‐2 (ATCC® HTB‐85) were used to produce cell‐derived extracellular matrices. Matrices were prepared, as described previously (Oliver‐Kozup *et al.*, 2013). Briefly, CAFs and Saos‐2 cells were cultured in high‐glucose Dulbecco's Modified Eagle Medium with 10% of fetal bovine serum and 1% of penicillin and streptomycin at 37°C in an atmosphere of 5% of CO_2_ throughout the experiment. To produce the extracellular matrix for specific tests, cells were grown as follows: (i) for matrix characterization via immunofluorescence or for GFP expressing GAS (GFP‐GAS) attachment assays, cells were grown on 15‐mm glass coverslips inserted into wells of (24‐well) tissue culture plates and (ii) for crystal violet biofilm assay, matrix characterization by ELISA or by Ponceau S staining, cells were growth in plastic wells without glass coverslips. Cells were seeded at 50,000 cells per well, grown until confluent and then detached through treatment with 5 mM of ethylene glycol tetraacetic acid (EGTA) and removed from wells. Samples were washed gently with PBS and wells or coverslips were subsequently used for assessment.

### Cell‐derived ECM matrix characterization

#### Ponceau S staining

To visualize the matrices deposited by CAFs and Saos‐2 cells, Ponceau S solution (0.1% in 5% of acetic acid solution) was added to the wells for 20 min. Stain was then removed, and wells were examined microscopically using a Zeiss Axiovert 40 CFL microscope with a 20× objective. Image acquisition was done using the Zeiss AxioCam Mrc5 camera and images analyzed with Zeiss AxioVision 4.8 software.

#### Matrix characterization by ELISA

CAFs‐ and Saos‐2‐derived matrices were prepared, as above and wells were denuded of cells and blocked overnight with 1% of bovine serum albumin in 1X TBS. The next day, the following mAbs were added to wells for 1 h at room temperature: anti‐fibronectin, specific for the fourth fibronectin type III repeat of fibronectin, (Sigma; IST4, 1:1,000) (Carnemolla *et al.*, 1983), anti‐EDA (Santa Cruz Biotechnology; IST9, 1:1,000)(Carnemolla *et al.*, 1983), anti‐EDB containing cFn (Sirius Biotechnology; C6, 1:500) (Balza *et al.*, 2009) or BC‐24, (1:1,000) (Nicolò *et al.*, 1990); a goat anti‐mouse secondary antibody conjugated with HRP (Jackson ImmunoResearch, 1:2,000) was added for 1 h, washed and then developed with ABTS substrate. Each antibody was used in triplicate wells, over three different experiments. Wells with secondary antibody only were used for background correction. Specificity of the primary anti‐ECM antibodies above, was additionally reaffirmed here through western blot analysis of commercial cFn (Sigma). Briefly, 1 µg of cFn was separated by SDS‐PAGE and transferred to a nitrocellulose membrane. Primary antibodies (BC‐24, 1:1,000; IST‐9, 1:500; IST‐4, 1:1,000; and C6, 1:1,000) were added, followed by secondary antibody horseradish peroxidase‐conjugated anti‐mouse IgG (H+L – 1:1,000). Blots were developed using Pierce™ ECL western blotting substrate (Thermo Scientific). Images were acquired using a ChemiDoc Touch Imaging System (Bio‐Rad).

#### Matrix characterization by immunofluorescence microscopy

To visualize the matrices deposited on glass, coverslips were blocked overnight at 4°C with 1% of BSA/TBS. Anti‐ECM antibodies: IST4 (1:100), IST9 (1:200), C6 (1:100) and BC‐24 (1:1,000) were added to wells for 1 h at room temperature, washed with TBS, followed by addition of goat anti‐mouse secondary Ab conjugated with Alexa Fluor 568® (Thermofisher, 1:300). Coverslips were then washed and mounted in ProLong Gold (Invitrogen). Matrices were visualized using a Nikon A1R confocal microscope equipped with a 60× objective. Images were processed using Nikon NIS‐Elements Software. BSA background images were each monoclonal antibody were taken and secondary‐only control images on cancer‐associated fibroblast‐derived ECMs were also carried out.

### GAS attachment and biofilm assays

#### Scl1 surface expression and rEDB binding by flow cytometry

Determination of Scl‐surface expression by GAS cells, as well as rEDB binding to whole‐GAS cells were measured by flow cytometry. Bacteria were grown to an OD_600_ of 0.5, harvested by centrifugation and washed with flow cytometry buffer (phosphate‐buffered saline containing 10% of Todd‐Hewitt broth with 0.2% of yeast extract). For Scl‐surface detection, the anti‐Scl1.1‐V antibody (Lukomski *et al.*, 2000), pre‐absorbed with MGAS5005 Δ*scl1* cells, was incubated with GAS cells for 30 min on ice. Cells were next washed and incubated with Allophycocyanin (APC)‐conjugated donkey anti‐rabbit antibody (Jackson ImmunoResearch; 1:150). For ligand binding, GAS cells tested were incubated with rEDB for 30 min at room temperature. GAS cells were then centrifuged and washed twice with flow cytometry buffer. Bound rEDB was detected with anti‐His‐tag mAb (1:750), followed by a goat anti‐mouse secondary pAb conjugated with Alexa Fluor® 568 (1:150). Cells were washed, fixed in 0.4% of paraformaldehyde and analyzed. 50,000 events were collected per sample using a BD LSRFortessa flow cytometer and data were analyzed with FCS Express Flow 6 software.

#### GAS whole‐cell attachment assay

GFP‐GAS adherence was studied on glass coverslips with rEDB coating and on cancer cell‐derived matrices, as described (Caswell *et al.*, 2010). 1 ml of GFP‐GAS cultures, prepared as above, were seeded into wells and incubated for 1 h at 37°C, washed with PBS, fixed with 3% of paraformaldehyde for 30 min and then mounted in ProLong Gold overnight. The total number of GAS cells were counted in 20 randomly selected fields and the average number of WT M1 or M41 GAS cells were set as 100%. The differences between WT and isogenic Δ*scl1* mutants were evaluated statistically. For inhibition assays, WT M41 GAS were pre‐incubated 30 min prior to attachment to CAF‐derived matrices with rEDA, rEDB, rTnFn1‐5, rTnFn6‐8 or rTnFn3. Cells were then added to CAF‐derived ECM for 1 h, washed with PBS and mounted as described above. The total number of GAS cells were counted in 30 randomly selected fields and the average number of WT M41 GAS that was not pre‐incubated with any ligand was set as 100%. The differences between treated and untreated GAS cells were evaluated statistically.

#### Crystal violet staining of GAS biofilms

GAS biofilm formation was tested on rEDB‐coated surfaces and on cancer cell‐derived matrices. Wild‐type GAS strains, their isogenic Δ*scl1* mutants, as well as complemented mutant stains, were grown to an OD_600 _of 0.5 and 1‐ml aliquots were seeded into 24‐well culture plates coated either with rEDB (2 µg/well) or in wells containing CAF‐ or Saos‐2‐derived ECM, prepared as above. GAS biofilms were grown for 24 h, washed with PBS and stained with 1% (v/v) crystal violet solution (Becton Dickinson) for 30 min at room temperature. Biomass staining was solubilized with 0.5 ml of 90% of ethanol and assessed spectrophotometrically at OD_600 nm_.

#### Confocal laser scanning microscopy of GAS biofilms

GAS biofilms were also visualized on glass coverslips coated with rEDB (2 µg) or on cancer cell‐derived matrices, using isogenic GFP‐GAS (Caswell *et al.*, 2010; Oliver‐Kozup *et al.*, 2011). The 24‐h biofilms were grown as above, fixed in 3% of paraformaldehyde for 30 min and mounted in ProLong Gold overnight. Biofilms were imaged using a Nikon A1R confocal microscope, with a 100x objective. Images were analyzed and deconvoluted using NIS‐Elements Software. Conversion to three‐dimensional images was performed with conventional Z‐stacks, deconvoluted stepwise and transformed using NIS‐Elements Software.

#### Statistical analyses

Statistics were performed using the two‐tailed paired Student's *t*‐test, one‐way and two‐way ANOVA, pending the experiment. Significance was denoted at levels of *P ≤ 0.05, **P ≤ 0.01 or ***P ≤ 0.001. Error bars represent standard deviations with analyses based on three independent experimental repeats (*N* = 3), each performed in triplicate technical replicates, unless otherwise noted.

## Conflicts of interest

The authors report no conflicts of interest with the contents of this article.

## Author contributions

DHM and SL contributed to the design and conceptualization of the study; DHM, SJC, JLA, RAH, SAW, LVDW and RB, provided reagents, performed experiments, acquired and analyzed the data; DHM, LVDW, RB and SL interpreted the data; DHM and SL wrote the original draft of the manuscript.
