# Supplementary material for: Adaptation of the group A Streptococcus adhesin Scl1 to bind fibronectin type III repeats within wound‐associated extracellular matrix: implications for cancer therapy
Source: Mol Microbiol. 2019 Jun 12;112(3):800–19. doi: 10.1111/mmi.14317 (PMC6736723; doi:10.1111/mmi.14317)
Supplement: Supplementary file 1 [file MMI-112-800-s001.pdf]

Adaptation of the group A *Streptococcus* adhesin Scl1 to bind fibronectin type III repeats within wound-associated extracellular matrix: implications for cancer therapy

Dudley H. McNitt[1], Soo Jeon Choi[1], Jessica L. Allen[2], River A. Hames[2], Scott A. Weed[2], Livingston Van De Water[3#], Rita Berisio[4#], Slawomir Lukomski[1*]

[1]Department of Microbiology, Immunology, and Cell Biology, West Virginia University School of Medicine, Morgantown, WV, USA
[2]Department of Biochemistry, Program in Cancer Cell Biology, West Virginia University School of Medicine, Morgantown, WV, USA
[3]Departments of Surgery and Regenerative and Cancer Cell Biology, Albany Medical College, Albany, NY, USA
[4]Institute of Biostructures and Bioimaging, National Research Council, Naples, Italy

[#]Both authors equally contributed to this work

***To whom correspondence should be addressed:** Department of Microbiology, Immunology, and Cell Biology, West Virginia University School of Medicine, 2095 Heath Sciences North, PO Box 9177, Morgantown, WV 26506, USA. Tel: +1 304 293 6405; fax +1 304 293 7823; email: slukomski@hsc.wvu.edu

Supplementary Information

Table S1

Sequences of Scl-V chimeras. Inserted loops are underlined and in bold. Sequence identities and root mean square deviations from Scl2.3 structure are given in the last two columns.

	Scaffold	Loop	Sequence of chimeras	Seqid (%)	R.m.s.d. (Å)
**Scl.chi1**	Scl2.28	Scl1.1	DEQEEKAKVRTELIQELAQ**KYPEVSNEKFWERKWYGTYFKE**LTYLQEREQAENSWRKRLLKGIQDHALD	35.8	1.1
**Scl.chi3**	Scl2.28	Scl1.28	DEQEEKAKVRTELIQELAQ**KEYPKASEEKFWESSFWGRRYFNE**LTYLQEREQAENSWRKRLLKGIQDHALD	35.4	2.5
**Scl.chi2**	Scl1.1	Scl2.28	EVSSTTMTSSQRESKIKEIEESLK**GLGGIEKKNFPTLGDEDLDHTYMTKL**EDFQKELKDFTEKRLKEILDLI	33.0	2.6
**Scl.chiC**	Scl2.28	Scl1.1	DEQEEKAKVRTELIQELAQGLGGIEKKNFP**ERKWYGTYFKE**LTYLQEREQAENSWRKRLLKGIQDHALD	40.3	1.1

Figure S1

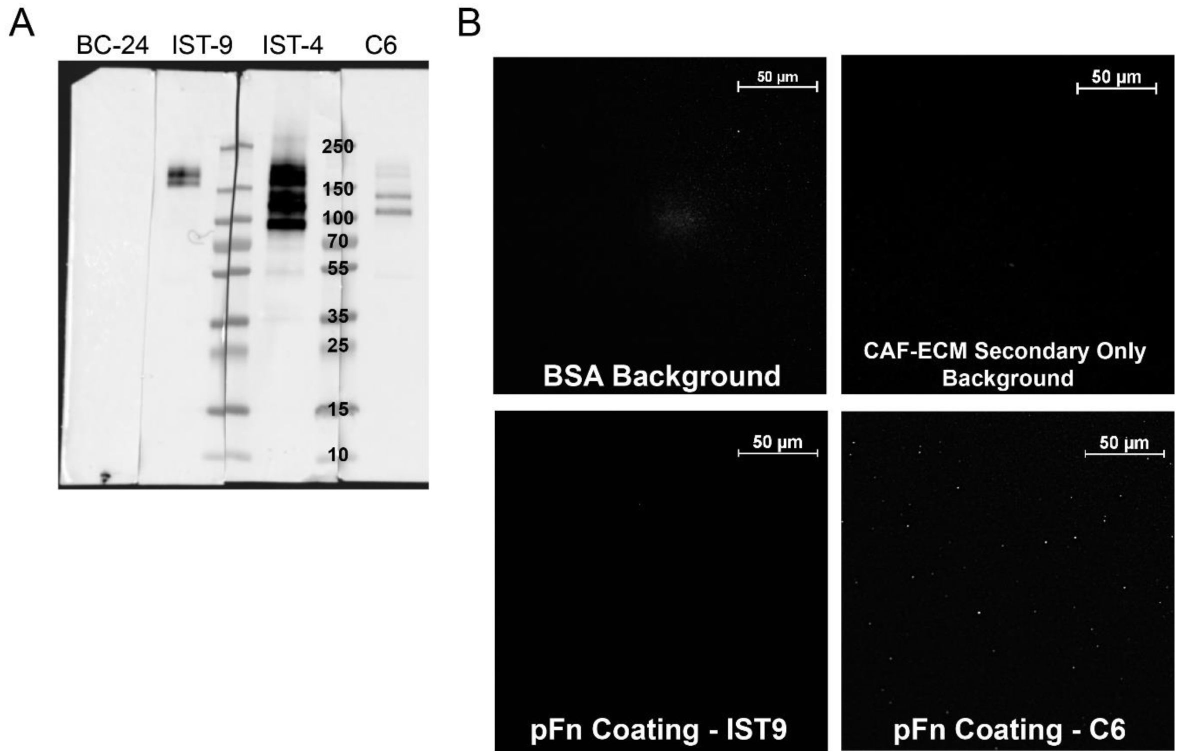

Fig. S1. Specificity of anti-ECM antibodies and background controls.

A. Antibodies against TnC (BC-24), EDA containing (IST-9) or EDB containing (C6) isoforms of cFn, as well as anti-fibronectin monoclonal antibody (IST-4), were analyzed by western immunoblotting of commercial preparations of cFn. Molecular weights, in kDa, are shown for PageRuler™ Plus protein ladder.

B. Immunofluorescent control images. 1 µg of BSA, 2 µg of plasma fibronectin (Sigma), or CAF-deposited matrices, were prepared on glass coverslips and incubated with primary mAbs, outlined in Fig. S1A. Secondary Ab conjugated with Alexa Fluor® 568 was used for detection. A representative image of BSA background (using anti-EDB antibody, C6) is shown in upper left panel, secondary only background on CAF-deposited ECM on upper right panel, and plasma fibronectin coatings in the bottom two rows. Images were taken using Nikon A1-R confocal microscope with 60x objective; representative images are shown from 2 independent experiments, imaging 10 arbitrary fields per coverslip.

Figure S2

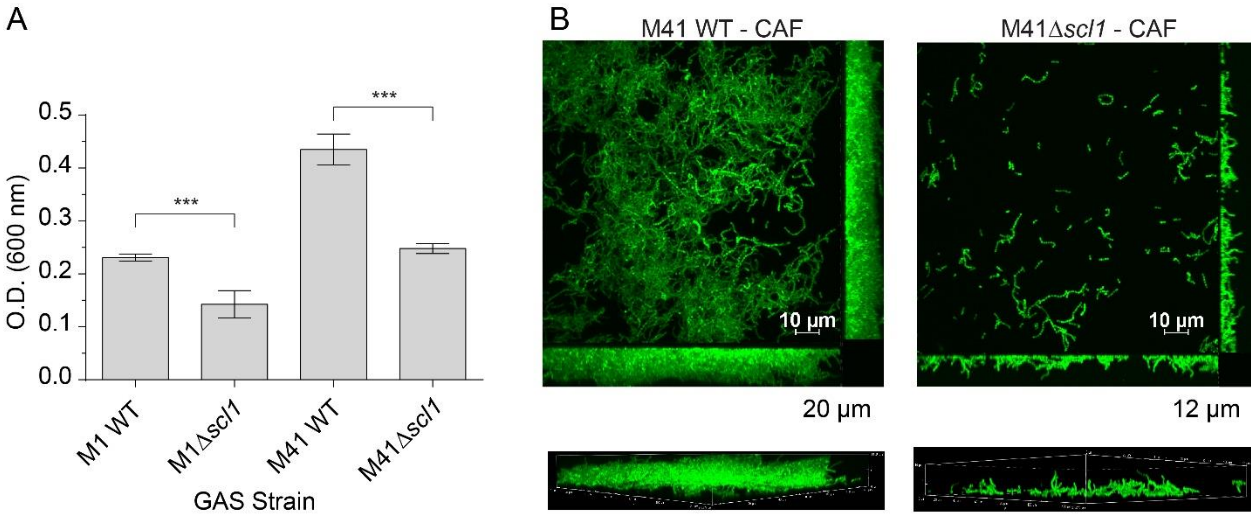

Fig. S2. Scl1-mediated GAS biofilm formation on ECM deposited by cancer-derived fibroblasts (CAFs). WT GAS strains M1 and M41, and their isogenic Δ*scl1* mutants were compared for biofilm formation.

A. Assessment of biofilm formation on rEDB-coated surfaces. M1 and M41 WT and their Δs*cl1* isogenic mutants were compared. Biofilm formation was evaluated spectrophotometrically following crystal violet staining. Graphic bars indicate the mean OD_{600nm} normalized against BSA controls. Statistical analysis was calculated using Student's two-tailed *t*-test from three independent experiments (N=3±SD); ***P≤0.001.

B. Microscopy imaging of GAS biofilms formed on CAF-derived ECM. GFP-expressing M41 WT and Δ*scl1* mutant strains were grown for 24 h on CAF-ECM matrix deposited on glass coverslips. Two-dimensional orthogonal views of GAS biofilms are representative of Z stacks from 10 fields within a single experiment. Average vertical thickness is indicated in micrometers below two-dimensional orthogonal views, taken from 10 arbitrary fields per experiment.

Figure S3

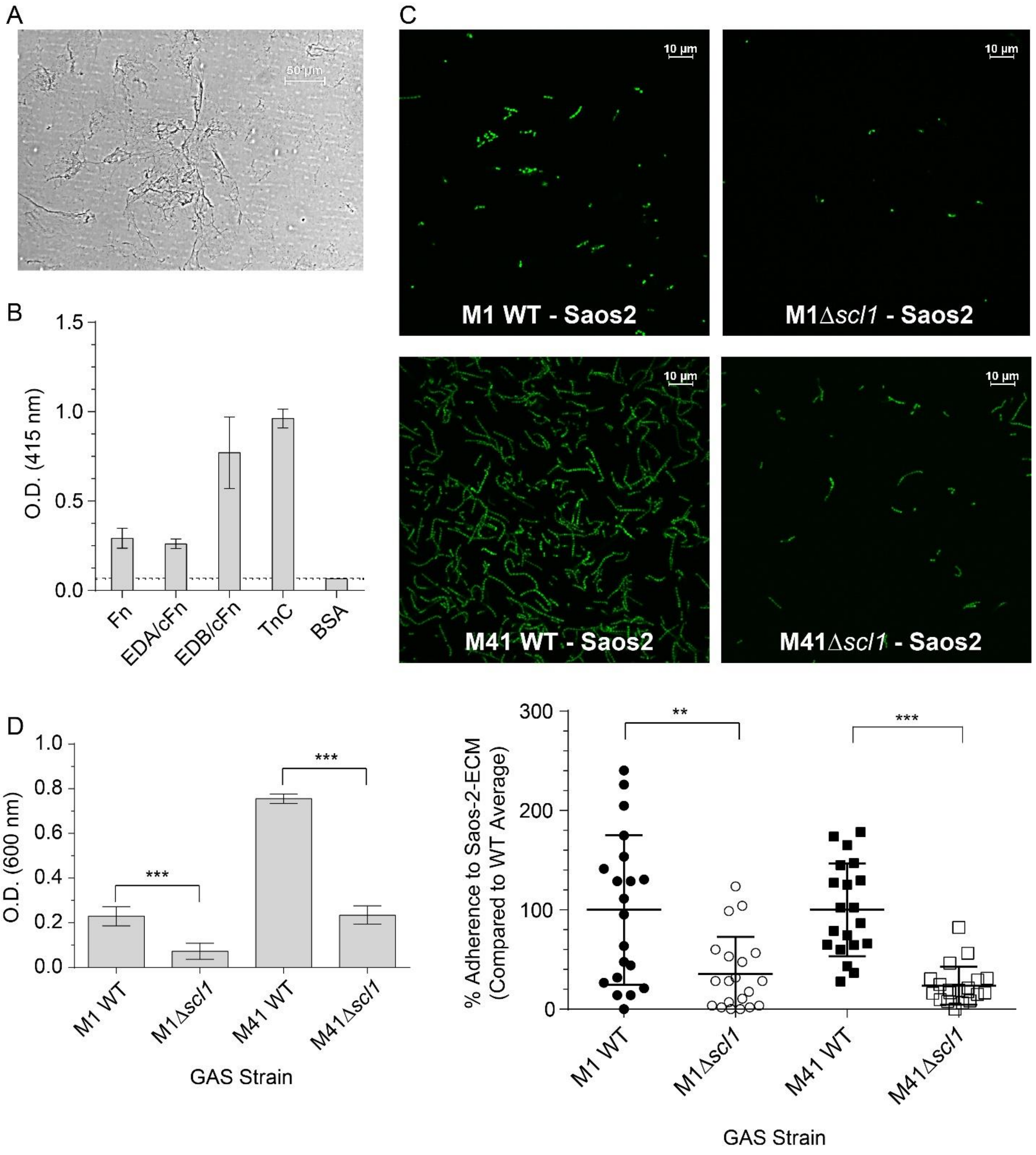

Fig. S3. Scl1-mediated GAS attachment to and biofilm formation on ECM deposited by bone osteosarcoma cells. WT GAS strains M1 and M41, and their isogenic Δ*scl1* mutants were compared for attachment and biofilm formation on the ECM produced by osteosarcoma Saos-2 cells.

A. Representative image of Ponceau S staining of ECM network deposited by Saos-2 cells.

B. Characterization of the ECM deposited by Saos-2 cells by ELISA. The presence of total Fn, EDA/cFn, EDB/cFn, and TnC was assessed with specific anti-ECM mAbs and secondary HRP-conjugated antibody. Graph bars indicate the mean OD_{415nm} from three independent experiments, each in triplicate wells (N=3±SD). Dashed line indicate threshold OD_{415nm} +2SD values recorded for BSA control wells.

C. GAS attachment on Saos-2-derived ECM. Isogenic GFP-GAS strains were inoculated onto Saos-2-derived matrices, allowed to attach for 1 h, and imaged using fluorescent confocal microscope with 100x objective. *Top,* representative images of attached GAS were taken in 20 fields. *Bottom,* quantification of GAS attachment with WT binding set as a 100%. Bacteria were counted in 20 fields and the average was calculated. Statistical analysis was calculated using Student's two-tailed *t*-test from two independent experiments, each performed in duplicate wells (N=2±SD); **P≤0.01, ***P≤0.001. Statistical significance evaluates the difference between adherence to Saos-2-derived matrices by the WT and their respective isogenic Δ*scl1* mutants. Each symbol represents one imaged-field.

D. GAS biofilm formation on Saos-2-derived ECM. Isogenic GAS strains were inoculated onto Saos-2-derived matrices and grown for 24 hours. Bacterial biomass was evaluated spectrophotometrically following crystal violet staining. Graphic bars indicate the mean OD_{600nm} normalized against BSA controls. Statistical analysis was calculated using Student's two-tailed *t*-test from three independent experiments (N=3±SD); ***P≤0.001.
